# Can you be Mindful? The Effectiveness of Mindfulness-Driven Interventions in Enhancing the Digital Resilience to Fake News on COVID-19

**DOI:** 10.1007/s10796-022-10258-5

**Published:** 2022-03-02

**Authors:** Padmali Rodrigo, Emmanuel Ogiemwonyi Arakpogun, Mai Chi Vu, Femi Olan, Elmira Djafarova

**Affiliations:** Newcastle Business School, Northumbria University, City Campus East 1, Newcastle upon Tyne, NE1 8ST UK

**Keywords:** COVID-19, Digital resilience, Fake news, Information management, Mindfulness

## Abstract

This study explores the factors that influence the dissemination process of and public susceptibility to fake news amidst COVID-19. By adopting a qualitative approach that draws on 21 interviews with social media users from the standpoint of source credibility and construal level theories, our findings highlight motives of news sharers, platform features, and source credibility/relatedness as major factors influencing the dissemination of and public susceptibility to fake news. The paper further argues that public susceptibility to fake news can be mitigated by building an integrated approach that combines a tripartite strategy from an individual, institutional and platform level. For example, educating the public on digital resilience and enhancing awareness around source credibility can help individuals and institutions reflect on news authenticity and report fake news where possible. This study contributes to fake news literature by integrating concepts from information management, consumer behaviour, influencer marketing and mindfulness to propose a model to help authorities identify and understand the key factors that influence susceptibility to fake news during a public crisis such as COVID-19.

## Introduction

The emergence of COVID-19 has given rise to the parallel phenomenon of infodemic where the spread of fake news increases as the pandemic continues to spread globally. Fake news can be defined as ‘news articles that are intentionally and verifiably false and could mislead readers’ (Allcott & Gentzkow, 2017, p. 213). Prior research has interchangeably defined fake news as either disinformation or misinformation (Di Domenico et al., 2021). While both dis-and misinformation include false content, disinformation involves the deliberate creation of false information with the intention to deceive and/or cause harm (Jack, 2017). Conversely, misinformation refers to the unintentional sharing of false information (Hernon, 1995). Different forms of misinformation have evolved due to the rapid growth of the Internet and technological advancements, and the resulting fake news is psychologically tailored (Au et al., 2021). And during a crisis, the spread of misinformation can lead to critical consequences (Abouzeid et al., 2021). In this paper, the term fake news is used as an umbrella term that encompasses both disinformation and misinformation, depending on the source and motive of information sharing.

Social media have become the ‘new public square’ where a wide range of information is disseminated and consumed. Social media are also the main platform through which fake news is shared (Rampersad et al., 2019). Since the outbreak of COVID-19 in early 2020, several fake news was spread on social media, including the origin of COVID-19 linked to 5G technology and dangerous medication. Building on the above definition, such information is often shared deliberately to mislead and/or cause harm (disinformation). However, in certain situations, fake news can be shared without the sharer knowing it is fake. Consequently, sources and sharers are often found to have a significant influence on the dissemination of fake news (Talwar et al., 2019).

The proliferation of fake news disseminated under the guise of news reporting (Fedeli, 2020) has made it difficult for individuals to recognise the credibility of such news. This has become even more difficult as such information is also shared by sources regarded as ‘credible’. Apart from the fake news shared by the public, sources perceived as credible (e.g., government officials, social media influencers and celebrities) have been accused of sharing fake news. This situation aligns with the source credibility (Hovland & Weiss, 1951) and construal level (Trope & Liberman, 2003, 2010) theories. While the source credibility theory suggests that the information shared by credible sources is likely to be believed as more people tend to engage with such information (e.g., via re-sharing, likes, and comments), construal level theory posits that the persuasiveness of a message is higher when the receiver experiences a small (as opposed to large) amount of distance and receives low levels of concrete (rather than high-level, abstract) persuasive messages (Nan, 2007). The latter is mainly due to the homophily that exists within an individual’s social media network, which, in turn, influences disparate believability of falsehood (Spohr, 2017).

Given the rise of fake news during the COVID-19 pandemic, national governments and international institutions are actively seeking ways to mitigate the negative effects of fake news and develop effective communication strategies to raise public awareness and digital resilience towards the damaging impact of false information. Prior research highlights several methods for combatting or pre-bunking fake news (Mele et al., 2017). Pre-bunking approaches include literacy interventions (Allcott & Gentzkow, 2017; Jang & Kim, 2018), fact-checking (Bernhard & Dohle, 2015) and detecting the direct origin of fake news (Ma et al., 2015). Nevertheless, these approaches are criticised for oversimplifying the issue as they seek to transfer responsibility to an ill-informed public (Mihailidis & Viotty, 2017). Therefore, it is imperative to develop an effective and robust digital resilience mechanism to mitigate the negative effects and individuals’ susceptibility to fake news.

Digital resilience helps individuals to recognise and manage the risks they face in online settings (GOV.CO.UK, 2021). The literature on information management identifies several ways to build (digital) resilience. However, these approaches fundamentally focus on educating consumers about digital safety. While such education is effective at the personal level, individuals can still be vulnerable to fake news. There is a need for an individual-level approach to developing strong resistance to fake news. Mindfulness can be a critical element in this regard, acting as an effective mechanism through which one can build digital resilience in a way that mitigates the susceptibility to the fake news shared by different sources.

To this end, we draw on the above discussions to propose two research questions (RQ) for this paper:

- RQ1: What factors influenced the dissemination process of and public susceptibility to fake news in the context of COVID-19?
- RQ2: What strategies could help to mitigate the impact of fake news on society?

We will integrate concepts from mindfulness, information management, construal level, and source credibility theories with how fake news was experienced by social media users during COVID-19 to answer these questions.

The remainder of this paper is organised as follows. Section 2 explores the theoretical background of the study, including the conceptualisation of source credibility, psychological distance, digital resilience and mindfulness. We then highlight the methodology adopted in Sect. 3 while Sect. 4 outlines the findings. Section 5 provides a discussion of the results and conclusions along with the study limitations and future research directions.

## Theoretical Background

### Fake news and the emergence of an infodemic

Social media are one of the major platforms through which individuals engage with each other (Kaur et al., 2021; Liu et al., 2021; Olan et al., 2022) and access information (Laato et al., 2020). Social media play a significant role in “*polarising views on politics, climate change, and more recently, the COVID-19 pandemic*” (Modgil et al., 2021, p.1). Social media are also used by governments and other authorities to provide real-time information to the public (Tran et al., 2021).

During the ongoing COVID-19, public engagement with social media increased markedly with a plethora of information shared across different social media platforms (ITU, 2021). Since the outbreak of COVID-19, a significant number of conspiracy theories, myths, rumours and information that generates mistrust in science has been shared online (Mukhtar, 2021; WHO, 2020). The spread of fake news not only generates negative consequences for society (Kaur et al, 2021; Modgil et al., 2021; Talwar et al., 2020) and governments (Allcott & Gentkow, 2017) but also has economic and financial consequences (Clarke et al., 2020; Visenti et al., 2019).

The proliferation of such fake news has resulted in the emergence of an infodemic that created a deep level of anxiety and confusion as individuals become increasingly fearful and circumspect about the authenticity of public information and its safety. Freckelton (2020) argues that the proliferation of falsehood has made people vulnerable to impulsive conspiracy theories about the causes of COVID-19 and how the various governments are responding to the pandemic. Consequently, individuals tend to believe misleading and unscientific information about dangerous medication, the origins of COVID-19 and governments’ responses. Prior research suggests that selective exposure or confirmation bias is one of the key determinants of consumer susceptibility to fake news (Kim & Dennis, 2019; Quattrociocchi et al., 2016) as individuals tend to believe information aligned with their ideologies (Lewandowsky et al., 2012). Furthermore, cognitive ability can also influence the susceptibility to fake news, specifically for individuals who are less analytic and gullible to fake news (Pennycook & Rand, 2019). As such, ill-informed and/or intentional malicious individuals could spread falsehood and exploit the public to create fear (Freckelton, 2020).

The responsibility for the spread of fake news can be ascribed to both human and non-human actors. Social and networked bots are considered key non-human actors responsible for spreading fake news on social media (Di Domenico et al, 2021). Social bots can accelerate the spread of fake news by making it viral (Azzimonti & Fernandes, 2018) and targeting influential social media users to include them in the spreading process (Shao et al., 2017). Human actors can also contribute to the spread of fake news as they may share falsehood deliberately or inadvertently if they align with their personal or ideological views (Di Domenico et al, 2021). Human actors may share or re-share content when they perceive the information to be trustworthy, credible and of high quality (Koohikamali & Sidorova, 2017). This behaviour becomes more prominent when the user has a high level of trust in the source or sender of the information (Talwar et al., 2019). Moreover, such sharing is influenced by the need to demonstrate conformity with other users. Hence, users spend little time and cognitive effort on information accuracy (Weinreich et al., 2008).

Therefore, the sharing of fake news by, for example, social media influencers is causing significant public disruption and negatively affects governments’ responses to the COVID-19 crisis. This is rapidly becoming problematic as governments and societies sometimes rely on social influencers and celebrities to combat fake news. For instance, similar to health care and grocery workers, the Finnish government classifies influencers as ‘critical actors’ during the COVID-19 pandemic (Heikkilä, 2020). Governments across Africa, Asia and Europe have also partnered with social media influencers to disseminate COVID-19 health information, specifically among younger audiences who are more susceptible to fake news to empower them to take appropriate official public health advice (Hutchinson, 2020). Consequently, it is important to understand the role played by sources perceived to be credible in the spread of fake news as source credibility has a significant influence on news credibility.

### Source credibility and construal level theories

The unprecedented spread of fake news via different social media platforms has made it extremely difficult for the public to assess news credibility and for governments to develop evidence-based decisions and strategies to tackle the pandemic (Erku et al., 2020). Vosoughi et al. (2018) found that false news spreads faster than true news via social media. Due to the ideological homophily of online platforms, social media users tend to perceive fake news as accurate and such information is shared faster between like-minded individuals (Spohr, 2017).

Source credibility can indicate whether the message or news communicated can be influential and impactful on the receiver. The main components of the source credibility model include trustworthiness, attractiveness and expertise (Hovland et al., 1953). When an individual has trust in the communicator, a message will be more influential and can change the opinion of the reader on a relevant subject. The expertise of the person communicating the message is also important as sources with lower expertise are perceived as less convincing (Erdogan, 1999; Ohanian, 1991). Perceived expertise is the most important aspect in terms of credibility for the public. This is particularly the case concerning medical advice or other important topics related to the pandemic. Perceived expertise can lead to positive attitudes towards investment and can influence purchase intentions (Seiler & Kucza, 2017). When the readers perceive the source as more credible, they form a more positive attitude towards the message (Eren-Erdogmus et al., 2016).

Source credibility theory is the most comprehensive concept that can be applied to the social media context because it focuses on the characteristics of the source (Djafarova & Rushworth, 2017; Ohanian, 1990). Fifteen sub-credibility factors have been widely accepted and used in the literature (Pornpitakpan, 2003; Sertoglu et al., 2014). These include attractiveness, expertise, knowledge, trustworthiness and reliability (Ohanian, 1991). In any case, the communicators of online messages affect message credibility, which then leads to changes in individual behaviour. High trust in online communicators reduces the level of message scrutiny by readers as people assume validity (Kareklas et al., 2015). Hence, when fake news is shared by a credible source, there are low attention levels to information accuracy and scrutiny.

Source credibility is also dependent on the quality of the argument and the persuasive strength of the endorser. Argument quality refers to the persuasion strength of arguments within an informational message (Teng et al., 2014). This suggests that when statements are perceived as valid on social media posts, viewers will have positive attitudes towards the product being endorsed relative to these messages (Spry et al., 2011). The quality of the message on social networking sites is supported by the strength of public perception of the source and elements such as relevance and timeliness. Relevance refers to the extent to which reviews are relevant and applicable (Teng et al., 2014). In terms of social media relevance, this could be due to specific issues related to those who communicated the message. For example, celebrities who have personally experienced mental health issues are perceived as more credible and relevant information sources when communicating content related to health. The originality of the source can further affect their perceived credibility (Casaló et al., 2020). Originality can also persuade others to act and change behaviours (Derbaix & Vanhamme, 2003). Messages/news shared by those that are similar to the viewers or perceived as trustworthy and knowledgeable is more likely to be believed regardless of their accuracy (Visentin, et al., 2019).

Construal level theory (Trope & Liberman, 2003: 2010) suggests that when psychological distance increases, individuals are likely to perceive objects/events in an abstract way (high-level construal) compared to a concrete way (low-level construal). Malär Krohmer et al. (2011) refer to this scenario as temporal distance. When using social media, individuals may be more likely to pay attention to information shared by close friends because of the psychological proximity, which is driven by factors such as a sense of belonging and homophily. Consequently, if a close friend shares any fake news, an individual will likely engage and believe it. Source credibility and the psychological distance of a source have a significant effect on how individuals perceive the credibility of the source and, by extension, the credibility of the news they receive and share. The psychological closeness between the sender and receiver could increase an individual’s susceptibility to fake news. Any fake news shared by a perceived credible source that is psychologically closer will be perceived as ‘true’. Thus, increasing the susceptibility to fake news and resulting in an overall higher level of susceptibility.

### Digital resilience and public susceptibility to fake news

Despite the unprecedented and far-reaching impact of fake news during COVID-19, there is a lack of robust policy guidelines to help authorities and the wider society facilitate better information processing. This study argues that digital resilience and mindfulness can act as critical building blocks for conceptualising an effective strategy to mitigate the impact of fake news on society. While the next section addresses mindfulness, this section explores the underlying information management mechanisms necessary for building a digitally resilient society. Digital resilience involves developing a formidable system that is reinforced with trust and integrity in a manner that adjusts/supports the disruption of online activities (Boh et al., 2020; GOV.CO.UK, 2021; Rai, 2020). As the impact of fake news continues to spread, digital resilience is critical in helping individuals manage the risks (GOV.CO.UK, 2021; Humprecht et al., 2020).

The dissemination of clear information and unambiguous communication are critical tools for managing crises such as COVID-19 (Arakpogun et al., 2020a). One of the major adverse effects of COVID-19 and the related infodemic is inefficient government messaging (Islam, et al., 2020; Mahase, 2021). The first proposed information management mechanism is intended to help authorities build a digitally resilient society through effective communication via multiple platforms (Arakpogun et al., 2020a; Baines & Elliot, 2020). Effective communication involves providing real-time relevant and reliable information via traditional and social media to keep the public abreast with developments (Baines & Elliot, 2020; Legido-Quigley et al., 2020; Kalsnes, 2018).

Digital literacy is an essential mechanism for reinforcing citizens’ resilience to misinformation and other forms of fake news (McDougall et al., 2019). Varying degrees of evidence on how the lack of digital skills hinders people’s ability to engage with technology abound globally (Arakpogun et al., 2020b). For instance, despite the availability of digital technologies for several decades, elderly members of society still experience difficulties in familiarising themselves with adopting digital tools and services (Vassilakopoulou & Hastad, 2021). Authorities, therefore, need to facilitate widespread informed usage of technology to build digital resilience. Informed usage involves ensuring that individuals have the relevant digital skills to use technology as well as online safety awareness (Arakpogun et al., 2017; Heeks, 2010). This raises the need for digital education (Burkhardt, 2017; Luttrell et al., 2020). It also encourages people to be sceptical about the authenticity of online information (Burkhardt, 2017; Guess et al., 2020).

Given the limitations inherent in the traditional medium of communication, scholars argue that it is apposite for authorities to include local community engagement in information management systems (Rai, 2020; Wright, 2016). Authorities typically communicate with the public through ‘official’ language. Such communication practices do not often account for local dialects, colloquial communications and the languages of minority groups or those living outside major cities (Arakpogun et al., 2020b). Therefore, relying solely on the official language for communication will lead to the exclusion of certain groups. Perpetrators of fake news often target the vulnerability of these excluded groups. These structural differences need to be acknowledged and addressed when formulating policy guidelines for building fake news resilience (Humprecht, 2020). Local community engagement helps mitigate the limitations associated with formal communication as information dissemination becomes localised.

### Mindfulness and information processing

The concept of mindfulness emerged from ancient Buddhism, where mindfulness is related to Buddhist concepts such as *sati* and *vipassana* in Pali (Wang et al., 2021). *Sati* refers to focusing on the present moment/object and *vipassana* refers to the deep observance of moment/object/behaviour (Hanh, 2014). Mindfulness is considered a key trait and has been recognised as a critical coping mechanism in online settings (Berthon & Pitt, 2019).

Mindfulness-based interventions are effective in reducing anxiety and stress (de Vibe et al., 2017). Extant research has employed the concept of mindfulness from different perspectives. Mindfulness as a trait reflects individual differences in consciousness, characterised by receptive attention to and awareness of present events, experiences without evaluation, judgment and cognitive filters (Glomb et al., 2011). Individuals with higher trait mindfulness tend to pay closer attention to the events happening around them (Brown & Ryan, 2003; Glomb et al., 2011). As these individuals can pay attention to experiences and events without judgment, they are also in a better position to deal with negative situations (Glomb et al., 2011).

Conversely, right mindfulness refers to the presence of mind based on capability experiences, which expand the breadth of attention and moment awareness to accumulate wisdom, experience and knowledge (Anālayo, 2010; Bodhi, 2011) for enhancing personal development and self-transformation (Purser & Milillo, 2015). Mindfulness can help individuals deeply feel and understand the dynamic multiplicity of organisational life experiences and beyond (Linstead & Pullen, 2006; Zanoni et al., 2010). It can thus facilitate non-simplified interpretations (Weick & Sutcliffe, 2008), and an understanding of the impermanent nature of phenomena (Vu et al, 2018). In communication, right mindfulness extends present-centred non-judgmental awareness as it involves the ability to attend to and retain a lived experience to cultivate context-sensitive and reflexive approaches to address phenomena (Dreyfus, 2011; Jha et al., 2010).

Mindfulness can help the public process information effectively as mindfulness enables the evaluation of new information in a manner that creates greater sensitivity to one’s environment (Bishop et al., 2004; Frauman & Norman, 2004; Langer & Moldoveanu, 2000; Van Winkle & Backman, 2008). Social media users often demonstrate homophilic behaviours as they find themselves in groups where users tend to follow the people, they are close to (Spohr, 2017). Within social media networks, homophily can result in the creation of coalesced attitudes, echo chambers and polarised networks (Shore et al., 2018). In such situations, individuals tend to be less mindful and less likely to check or assess the credibility of the information they share. Therefore, we argue that enhancing an individual’s level of mindfulness and using mindfulness-based interventions can be useful for encouraging people to develop their sensitivity towards the environment, be more aware of their actions, curtail cognitive laziness and enhance information processing (Wimmer et al., 2016).

### Prosocial behaviour, mindfulness and digital resilience

Mindfulness is not only associated with attentional capabilities but also with the ability to process others’ emotional experiences. This process is known as interoceptive awareness (Farb et al, 2007; Singer et al, 2009). Such awareness informs individuals to be aware, behave and respond attentively to others (Brown & Ryan, 2003; Condon, 2017). This facilitates prosocial and positive emotions in ethical approaches towards communication (Fredrickson, 2017; Lutz et al., 2008). Prosocial orientation further helps facilitate ethical approaches to the arena of speech and action (Greenberg & Mitra, 2015) for communicating information responsibly. Mindfulness can be thus used to augment individuals’ information processing in a manner that prompts increased self-control and subsequent positive behaviours (Brown & Ryan, 2003; Brown et al., 2007), ethical awareness, reasoning and conduct (e.g., Barnett & Vaicys, 2000; Douglas et al., 2001; Jones & Kavanagh, 1996; Singhapakdi et al., 2000; Valentine & Barnett, 2007). Since fake news is often created by organisations that deliberately want to mislead people, mindfulness can be an effective mechanism to enhance the practice of digital ethics associated with the creation and dissemination of authentic and credible content.

Local community engagement and the awareness of such ethical digital practices can be facilitated by mindfulness approaches. Building upon prosocial behaviours, local community engagement should reflect that the commitment to resilience is about concentration, complemented by mindfulness as the means to achieve insights for future actions, including dealing with information transparency (Weick & Putnam, 2006). Having a trait mindfulness can help individuals build digital resilience as they pay closer attention to the information they are being exposed to and form an awareness (Brown & Ryan, 2003; Glomb et al., 2011) to evaluate information. Therefore, we argue that mindfulness can be an effective means through which individuals can build digital resilience.

## Research Approach

A qualitative approach was adopted for this study to enable us to explore the complexity and sensitivity (Figueroa‐Domecq et al., 2015) of fake news in its natural settings (Orlikowski, & Baroudi, 1991), a subject that requires a deeper understanding (Silverman, 2020) and intersubjective sensemaking (Walsham, 2006). As such, the data collection (21 semi-structured interviews) and analysis process were guided by an interpretivist epistemology—that knowledge is socially constructed via shared meanings, languages and consciousness (Orlikowski & Baroudi, 1991; Richey et al., 2018). Semi-structured interviews were used due to the flexibility and spontaneity it offers (Jones et al., 2012). This approach allowed the interviewees to respond, “freely within their own frame of reference” (Hankinson, 2009, p. 104) as they narrated how they experienced fake news in the context of COVID-19.

Adopting a qualitative approach from an interpretivist perspective allowed us to further understand the news-sharing practices of social media users and their motives behind sharing fake news. Hence, adopting an interpretivist epistemic perspective, we explored the phenomena of interest (factors that influenced the dissemination process of fake news during COVID-19 and strategies to reduce public susceptibility) “acknowledging that all meanings and notions of truth are relative, varied and construed” (Lee & Tao, 2021, p.654).

### Pilot interviews: data collection and analysis

Before conducting the main study, a pilot study was conducted among five respondents to determine the appropriateness of the themes and interview questions (Bryman & Bell, 2015; Charmaz & Belgrave, 2002; Creswell, 2013). For example, the pilot study revealed that the participants ascribed all news that was not credible, reliable and fabricated as “fake news” regardless of whether it was a deliberate act or not. Hence, we decided to add a question to the interview guide to explore *how* fake news is defined by the respondents. The pilot respondents’ profiles are presented in Table 1.

**Table 1:** Pilot Interview respondents’ Profiles

Respondent	Age	Gender	Occupation
1	38	Female	Customer service executive
2	36	Male	Engineer
3	34	Female	Engineer
4	40	Male	Business owner
5	28	Female	Teacher

The pilot participants provided us with a range of examples highlighting the nature of information and fake news that they were exposed to during COVID-19. Most of the news was shared by a user on their social media platforms such a Facebook and WhatsApp. The interview guide was then revised by adding some questions to explore the nature and source of the message as well as the platforms through which they were shared. When asked about strategies to mitigate public susceptibility to fake news, the pilot respondents highlighted the importance of enhancing awareness, safety and the need to encourage people to be more vigilant and mindful when sharing off-and-online news. This aligned with prior research in Sect. 2 that suggested that digital resilience and mindfulness can be effective in reducing public susceptibility to fake news. Two questions were then added to explore the respondent’s knowledge of mindfulness and digital safety.

### Main interviews: data collection and analysis

An interview guide was developed based on four key themes derived from the discussion in Sect. 2 and the pilot study. These include (1) user engagement with social media during COVID-19, (2) awareness of the unprecedented spread of fake news during COVID-19, (3) sources and processes through which fake news was disseminated and (4) how to mitigate public susceptibility to fake news. The interview guide evolved throughout our study, enabling us to revisit some of our early interpretations as the interviews progressed (Krefting, 1991). See Appendix 1 for the final interview guide.

Due to the COVID-19 restrictions in the UK during the data collection, interviews were conducted via telephone and other online platforms (e.g., MS Teams, Skype and Zoom). During the interviews, the respondents were first asked to explain their views on social media engagement as the COVID-19 crisis unfolded. For example, interviewees explained the nature of the information they shared via social media regarding COVID-19. Building on these responses, the respondents were then asked to reflect on the sources through which they received information and provide their views on the perceived credibility of the sources, and what persuaded them to share that information with others. Next, the respondents’ mindful awareness was explored along with the concept of digital resilience for which they were asked to present their views on the extent to which mindfulness and digital resilience can help reduce their susceptibility to fake news.

A total of 21 interviews were conducted separately from the pilot study. Our respondents were 18 years old and above who regularly used social media with a diverse range of occupations from academics to business owners, healthcare workers, students and the unemployed**.** Snowball sampling technique was employed to recruit respondents beginning from our contacts who are using social media during the ongoing COVID-19. Successful respondents were then asked to recommend other potential participants that would be interested in contributing to the study (Parker et al., 2019).

A total of 21 interviews was deemed appropriate as saturation began to emerge after the 16th interview (see Appendix 3). Research that follows thematic analysis often focuses on data saturation, which is defined as ‘information redundancy’ or the point at which no new theme or code ‘emerges’ from data (Braun & Clarke, 2021). In our study, we determined saturation by assessing code and meaning saturation. For instance, Appendix 2 presents an overview of the new codes that emerged after each interview. We did not have any prior threshold to determine code saturation. Hence, in line with Hennink et al. (2017), code saturation was determined by code identification and prevalence. All codes and sub-dimensions were identified by the 16^th^ interview and no new code or sub-dimension emerged after the 16^th^ interview.

The meaning of saturation was assessed by following the approach suggested by Hennink et al. (2017). We first selected the key codes and listed the dimensions found in each interview in relation to individual codes. Appendix 3 presents an example of sample codes we traced and listing the various dimensions of each code that were identified by the interview. Meaning saturation was determined to occur at the last interview in which a novel code dimension is identified. This indicates that the saturation of different codes can be reached at different time points (Hennink et al., 2017). In our study, Appendix 3 indicates that our meaning saturation for different codes and different sub-dimensions were reached at different timepoints. However, as with the code saturation, the overall meaning saturation for all key codes was reached by the 16^th^ interview. Hence, no new codes or meanings were identified after this point. Please see Appendix 4 for examples of some quotes from the respondents where repetitions can be seen with no new code or meaning emerging.

However, of the 16 respondents interviewed, 12 respondents were female, and four respondents were male. Hence, five additional interviews were carried out with male social media users, after the 16^th^ interview to see if the gender of the respondents had any influence. However, no new codes or meanings were identified during these additional interviews. Therefore, a total of 21 interviews was deemed appropriate and no additional interviews were conducted.

The data gathered were analysed using the six-step thematic analysis procedure proposed by Braun and Clarke (2014). Accordingly, the first two authors independently analysed the data by (a) familiarisation, (b) generating initial codes, (c) identifying themes among the codes, (d) reviewing the themes, (e) defining and naming the final themes, and (f) producing the final report. For example, in the first phase of familiarization, all data gathered were transcribed by the two aforementioned authors who conducted the interviews and were familiar with the data (Riessman, 1993). The transcribed data were then read and re-read repeatedly for familiarisation while making summary notes of initial ideas for coding. Based on the summary notes, the two authors then moved to the second phase of thematic analysis that involved generating initial codes by coding the interesting features of the data systematically across the whole data set while collating / organising data relevant to each code. Once all relevant data were coded and collated, extracts were sorted into potential themes. This involved combining different codes and the identification of overarching themes and sub-themes. Four overarching themes emerged at the final stage in response to our research questions. These include the motive, source, platform and mitigating strategies. In relation to motive, two subthemes emerged: deliberate vs mistake; in relation to source, two subthemes were also identified, namely, credibility and relatedness. Four subthemes emerged in relation to mitigating strategies—enhancing awareness, educating on digital resilience, empowering people with mindfulness and engaging with social media users.

**Fig. 1:**
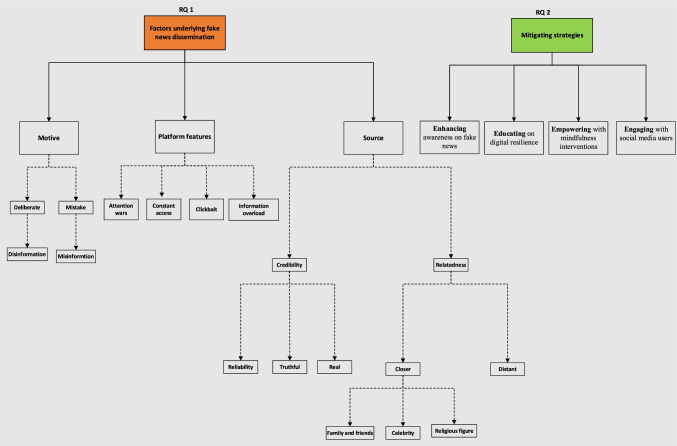
Fake news thematic data map

Figure 1 maps the overarching themes that emerged from the data analysis concerning the two research questions set out in Sect. 1.

## Findings

### The dissemination process of fake news

Figure 1 indicates that the major factors that accelerated and influenced the dissemination process of fake news during COVID-19 can be explained by motive, platform features and source.

#### Motive

Figure 1 indicates that when it comes to understanding the news sharing practices of social media users and their motives for sharing fake, participants suggested that two issues are critical – *deliberate* versus *mistake*. In order words, do people share fake news with a deliberate intention to harm or is such sharing done by mistake due to ignorance? Our findings revealed a mixed answer. For example, while some participants believed that fake news is often shared by the original creator or a given source to cause harm and/or mislead the public, other participants posited that fake news can be shared by mistake with people in the same social networks without the sharer realising it is fake. Participants also suggested that the motive for sharing fake news can both be deliberate and mistake. This is highlighted by the following:

*It can be both: they can have their own agenda and it can be by mistake. I am certain that there are individuals and organisations that do this on purpose… (RP-*6)

*I am convinced they do it deliberately. I supposed someone who is just forwarding messages they got from elsewhere could be doing it innocently. But when you think about these gossip papers who originated the news, they do it deliberately (RP14)*

Participants further attributed the motive behind deliberate fake news sharing to financial benefits, intention to cause harm/mislead the public and for political gains. This is partly highlighted below:

*…there are two groups involved. One is the group that aims to use fake news to generate money, to generate profit or to generate specific benefits for themselves. Others do it by manipulating people via spreading fake news to change people’s mindsets. Such people want to take advantage of people and they know they are doing something wrong, but they are just greedy and do not care about others except themselves (RP-13)*

Concerning those who share fake news by mistake, participants suggested that ignorance, genuine desire to inform loved ones, pressure to comply with one’s social circle and the intergenerational gap can be the reason behind sharing falsehood. Take the issue of the intergenerational gap, for example:

*I think the intergenerational divide has a role to play here. The younger generation was born into a different time where … social media platforms like Facebook are mainstream … it is easier for them to access Facebook than turn on a television … For my generation … our residual still empowers us to know what genuine stuff looks like … We still have an appreciation for authenticity but not so much for the younger generation. (RP-1)*

*I feel the susceptibility to fake news is more common with the older generation. And this vulnerability may not be due to the lack of knowledge or education; I just think they came from a generation where the news was from sources like a newspaper. (RP-6)*

While participants under 35 years old argued that the older generation was more gullible and culpable to believing and sharing fake news, participants over 35 objected to this idea, with a few exceptions saying age was not a critical factor but it was a matter of common sense and life experience whether one mistakenly share fake news or not. While there was no clear understanding of *which* age group is more likely to easily believe fake news, it was implied that the age of a social media user plays a role in public gullibility and susceptibility to fake news.

#### Platform features

`While participants generally agreed that social media platforms were useful tools during COVID-19 restrictions, platforms related attributes (e.g., attention wars, clickbait, constant access and information overload) exacerbated public gullibility and susceptibility to fake news. Take constant access and information overload for example. Since participants engaged with social media platforms (e.g., Facebook, Instagram, and WhatsApp) to stay connected with families and friends due to COVID-19 restrictions, they were constantly exposed to an avalanche of COVID-19 news and information – which the majority of participants went on to share with their social network. Most participants voiced their disquiet about the explosive volume of COVID-19 information (e.g., government initiatives, cures and treatments, vaccination programmes) on various social media platforms as the pandemic evolved. For instance:

*There was too much information shared across different social media platforms. It was overwhelming. Every second there was something new or an update. I found it a bit overwhelming and stressful. (RP -18)*

`Since constant access to social media markedly exposed the public to COVID-19 news and information, it became extremely difficult for people to differentiate between fake and real news. This led to an information overload and increased public susceptibility to fake news. Therefore, information overload was one of the platform features that was found to have a significant impact on how respondents perceived the authenticity, credibility and transparency of news information as highlighted below:

*I think a lot of people find it difficult to differentiate between what is real and what is fake. There is too much information from different sources. This makes it harder. (RP-21)*

In addition to platform attributes such as attention wars and clickbait, participants further revealed that easy access and the ability to quickly create social media accounts jointly contribute to the rapid spread of fake news.

#### Source credibility and relatedness

Section 2 indicates that sources (i.e., originators and/or sharers of news and information) are often found to have a significant influence on the dissemination of fake news. Similarly, participants revealed that they were mainly exposed to fake news via messages and posts from social media acquittances, individuals within their social circles and close relatives. The *relatedness* (close or distant) of a social media user to a source thus emerged as one of the key factors to the dissemination of fake news as indicated in Fig. 1. Furthermore, most participants opined that they perceive news shared by a related (close) source as *credible* and proceed to engage with such news/messages by liking or re-sharing to show their affection and conformity. As such, the engagement with news/messages from related sources that are perceived to be credible can also increase the spread of fake and public susceptibility. For example:

*…because the sharers believe and trust the original source, they just go ahead and circulate without verifying, with the mindset that their close contacts cannot mislead them or cannot be in the wrong. (RP-8)*

Some respondents further mentioned that within their families, there were people who shared *“…absolutely anything and everything…”* without checking the authenticity of what they were sharing. Consequently, close sources that are perceived as credible are super-spreaders of fake news via, for example, family WhatsApp groups. Many respondents further acknowledged that as the first-line recipients of fake news from their social circle, they may have shared and re-shared falsehood unknowingly without any intention to cause harm (misinformation).

Overall, our findings indicate that the psychological closeness between the respondent and the source can influence the former to share the news that they later realised to be fake. In such situations, most participants found it difficult to check whether the information was true, as it came from a source that they trust or have a close connection with.

### Strategies to mitigate public susceptibility to fake news

As for the potential mitigating strategies to limit the impact of fake on society, Fig. 1 outlines four strategies—*enhancing* awarenes*s* on fake news, educating the public on digital resilience, empowering people with mindfulness interventions and engaging with social media users. These three strategies emerged from our interactions with participants as they reflected on how they experienced fake news during the first COVID19 lockdown.

#### Enhancing awareness of fake news

Our findings indicate that from an institutional level, policymakers can enhance public awareness on the real-life dangers of fake news, how it is spread, why people fall victims and share them, and the different types of dis-and-misinformation. Such awareness can be raised via campaigns on social media and other traditional media using informed data in collaboration with relevant experts and reputable public figures with credibility. Interestingly, participants highlighted that such awareness should be raised across the ethic and intergenerational divide:

*For me, it’s all about awareness. I’ll give you an example, at the heat of the pandemic, there was fake news circulating that ethnic minorities such as those from Africa and Asia living in the UK have resistance to COVID-19. As such, people from these communities fell for this falsehood and began taking less precaution... But we now have data to show that actually more ethnic minorities died from COVID-19 in the UK compared to other ethnicities. So, you see the problem? If the society become more digitally resilient, they would ask for data to back such claim. (RP-8)*

As for the society, there should be increased awareness of the impact of fake news for both the older and younger generations…(RP-10)

#### Educating the public on digital resilience

Section 2 highlighted that digital resilience helps individuals identify the risks in online settings with a view to taking necessary actions to ensure safety. However, many respondents highlighted that they have little knowledge about recovery processes if they become a victim of fake news. For example:

*I wouldn't even know how to go about looking for recovery sources if I was a victim of fake news. I also wouldn't even know how to describe if someone is a victim of fake news. (RP-12)*

Therefore, our interactions with the participants highlighted the importance of educating the public on various aspects of digital resilience, including digital safety, human–computer interactions and how to engage on social media safely on an individual level. From an institutional level, participants suggested that governments should embed the concept of digital resilience into the education curricula at all levels to raise awareness of the dangers of fake news and build a pent-up societal resilience in a post-pandemic world. A pent—up increase in digital resilience will also go a long way in helping social media users overcome the difficulties associated with recognising fake news sophistication as participants acknowledged this as a challenge:

…*but I am aware that the people who are spreading and starting fake news are becoming a lot more sophisticated and cleverer in the way they are coming across. (RP-15)*

#### Empowering the public with mindfulness

Prior research has suggested that mindfulness can act as a medium for strengthening resilience. As such, participants’ perceptions of and to what extent mindfulness can be used to mitigate the effect of fake news were explored. Participants highlighted how mindfulness facilitates consciousness and cognitive awareness to process information that reflects a powerful means to fight against fake news:

*…being aware of the environment will give us the consciousness to do a bit of verification or seek additional help from friends, neighbours or the local community and if these additional sources can’t help to verify it, then there’s every likelihood that this may be fake news. (RP-6).*

The data further indicated that mindfulness also encouraged prosocial orientations through professional and personal ethics in information processing and sharing. Participants highlighted the importance to manage information processing at the community level, which indicates an awareness of prosocial behaviour to raise ethical awareness and reasoning behaviours to identify fake news. This position is reflected in the following excerpts:

I think mindfulness can help to limit the impact of fake news on society. (RP-3)

Government and organisations have some legal responsibilities to be mindful of the accuracy of the information they put out. All this has to do with professional and personal ethics by making sure you verify a piece of information before sharing it. (RP-4).

We observed that the tendency to be mindful appeared to be higher among participants who were previously victims of online fraud. Such participants possessed a certain form of trait mindfulness from previous experiences to enable them to pay closer attention to the events happening around them. Our findings further suggest that mindfulness can help inform individuals’ susceptibility to fake news by empowering people to be more aware of what is happening around them. To this end, participants argued that mindfulness can enhance their digital safety and resilience as it allows them to identify risks and decide on appropriate actions in an online setting. Overall, participants opined that while building digital resilience through education can be useful in reducing public susceptibility to fake news, mindfulness can help the public to build digital resilience by enabling people to be more aware of the authenticity of what they consume in the digital environment.

#### Engaging with social media users

While most participants acknowledged that it is a difficult task for social media firms to monitor the authenticity of every piece of news/information on their platforms, there was a general census that more can be done to engage with social media users. For example, respondents mentioned that it is extremely important to develop a two-way dialogue between social media platforms and users to mitigate the public susceptibility to fake news. More specifically, the participants underlined the importance of encouraging members of the public to report fake news while platforms must ensure they provide feedback/updates to the public on what actions they have taken on the reported cases. Some participants expressed their frustrations at platforms that do not engage with users:

*…there has been a lot of times when I have reported certain pages and certain things, but Instagram never really gets back to you about what they have done with the report you made, which is quite frustrating. I think it would be useful for Facebook and Instagram to feedback to people who reported certain sites, so you know what they have done with the information you provided them. (RP-14)*

Participants want social media platforms to be more transparent and proactive in their actions to encourage individuals, private organisations and governments to report fake news. For example, it was suggested that social media firms can create *source code verification* for consumers to verify the authenticity of information before the platform allows them to read and share such information.

Overall, our findings in Sect. 4.2 highlight the need to take an integrated tripartite approach at an institutional, individual and social media platform level to raise awareness and educate the public on fake news, its dissemination process and actions that can be taken to limit public susceptibility.

## Discussion and Conclusions

The COVID-19 crisis has dramatically increased the global spread of fake news, with detrimental consequences encompassing lives and livelihoods. Hence, it has become imperative for governments, international organisations, and social media firms to find effective ways to mitigate the spread of fake news with a more human focused approach (Di Domenico & Visentin, 2020). The World Health Organization (WHO) has called for member states to develop and implement action plans to better manage fake news, particularly among high-risk and vulnerable groups (WHO, 2020). Against this backdrop, this research set out to uncover w*hat* factors influenced the dissemination of fake news in the context of COVID-19 and *what* can be done to mitigate its impact on society.

Our findings indicate that the dissemination of fake news is mainly influenced by issues around motive, platform features and the perceived relatedness between the source/sharer and receiver of news/messages. As people spent more time on social media during the first lockdown of COVID-19, their exposure to and sharing of news exploded. This made it extremely difficult to differentiate between fake and real news. An increase in social media engagement combined with platform-related attributes (e.g., attention wars, clickbait, constant access and information overload) exacerbated public gullibility and susceptibility to fake news. Such findings complement the findings of previous studies (e.g., Allen & Shoard, 2005; Hermida, 2010; Holton & Chyi, 2012), which found that relentless access to social media exposes people to an increased volume of news and information overload.

Regarding the nature and motives behind the spread of fake news, respondents argued that fake news can be disseminated deliberately or by mistake. Specifically, organisations were seen as the most deliberate perpetrators (disinformation), while individuals largely share fake news without having a deeper understanding of the authenticity and impact of falsehood (misinformation). Hence, in contrast to the definitions in the literature that often define fake news as disinformation, the evidence from our study revealed that fake news is better defined as an embodiment of both dis- and misinformation, especially for fake news shared on social media platforms. This supports our proposed definition in Sect. 1 as originators may share with a deliberate attempt to mislead for personal gain, but the receivers often share by mistake or a false sense of understanding that they are looking out for their loved ones. Some participants argued that even if they know that their friends or people within their social media network would not harm them intentionally with false information, this does not mean that they might not unknowingly share fake news with them as experienced by many respondents during the COVID-19 pandemic.

Traditionally, source credibility is determined based on the trustworthiness, knowledge, expertise and attractiveness of the source (Hovland et al., 1953). However, our findings indicate that source attractiveness does not play a major role in how members of the public perceive source credibility compared to expertise and trustworthiness, which had a more significant influence. Interestingly, we found that the psychological closeness between the source and receiver influences the way respondents perceive the quality of news. In addition to the news received via media that are perceived to be credible and have a high level of reputation, news information received from a friend, or someone perceived as psychologically closer was perceived as credible and trustworthy. Hence, participants paid less attention to the credibility of the shared message, which then accelerated the dissemination of fake news across their social networks. This position complements and updates that of Rogers (1995), who argues that a source can be perceived as credible when there is a strong tie between the sender and receiver. As such, news shared by a source with a strong tie or psychotic proximity is perceived as credible (Coleman, 1990).

Therefore, we make a case for psychological closeness to be included as a dimension in the source credibility literature. As previously mentioned, news and information overload were key factors that made it difficult for the public to pay adequate attention to news and information accuracy by making it difficult to judge the reliability, authenticity, and accuracy of news (news credibility) on social media, as well as the credibility of the source (expertise, trustworthiness, and attractiveness of originators and senders). Participants argued that, although they tried to pay attention to news received from credible sources (e.g., the BBC, CNN), they found it difficult to assess credibility. This is particularly evident when individuals receive such information via a source that is perceived to be psychologically closer or related. This finding is consistent with Lee, Lindsey, et al. (2017), Lee, Lee, et al. (2017)), who argue that information overload can also have a significant influence on how people perceive news quality and source credibility.

**Fig. 2:**
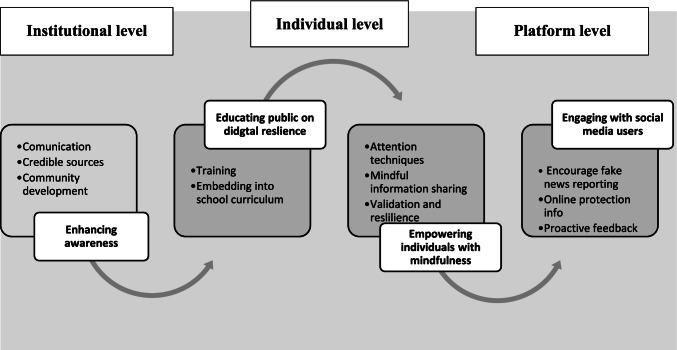
A tripartite model for mitigating the impact of fake news

Our interactions with participants highlighted the need to develop strategies at institutional, individual and platform levels to mitigate the negative impact of the fake news and limit public susceptibility. As such, we draw on our findings in Sect. 4.2 and argue that the impact of fake news can be mitigated by building an integrated approach that combines a tripartite strategy from an individual, institutional and platform level. Figure 2 summarises the elements within this tripartite model and for the sake of simplicity, we categorise them as 4Es: *enhancing* public awareness on fake news, *educating* the public on actions they can take to build resilience, empowering the public with mindfulness-driven interventions and engaging with social media users.

Take the institutional level, for example. While unambiguous communication and the dissemination of clear information are critical tools for managing the COVID-19 crisis (Arakpogun et al., 2020a), one of the adverse effects of COVID-19 infodemic is inefficient government messaging (Dearden, 2020; Islam, et al., 2020; Mahase, 2021). Participants argued that policymakers can enhance public awareness of fake news through constant and effective communication on multiple platforms. Such communication also needs to be timely to proactively counter fake news and control the narrative. At the individual level, participants highlighted the need to enhance digital skills and mindfulness. Being mindful facilitates reflexivity (Vu & Burton, 2020) with cautiousness and self-control in information processing and encourages prosocial orientations through professional and personal ethics in information sharing. Participants further stated that empowering the public with mindfulness can help enhance digital resilience by raising awareness and greater context sensitivity in information processing and sharing to identify risks and decide on appropriate actions in an online setting.

These findings complement and advance extant literature on the influence of mindfulness on awareness (e.g., Brown & Ryan, 2003; Glomb et al., 2011) since individuals with higher mindfulness tend to pay closer attention to and have an awareness of events happening around them. This is consistent with other studies (e.g., Bishop et al., 2004; Frauman & Norman, 2004; Langer & Moldoveanu, 2000; Van Winkle & Backman 2008), who argue that mindfulness allows individuals to further enhance their attitudes and perceptions. We thus argue that mindfulness and mindfulness-based practices can help improve digital resilience and the wider effort of reducing the spread of fake news.

Overall, this study contributes to the emerging body of knowledge on fake news and social media by identifying the factors that influenced the dissemination process of fake news during COVID-19. We further contribute to the discourse around consumer susceptibility to fake news by exploring the roles played by the source, psychological distance between the source and the receive and the effectiveness of digital resilience and mindfulness-based interventions in mitigating the impact of fake news. This study also contributes to the body of literature on fake news in the information systems domain by integrating theories from marketing and psychology to understand the role played by the source and source relatedness in the dissemination of fake news during the COVID-19 pandemic. Most of the prior research suggests that approaches such as education are needed to enhance the digital resilience of the public. We corroborate this and further argue that in addition to using institutional level strategies such as the provision of education, more attention should be focused on individual (e.g., empowering individuals with mindfulness) and platform levels (e.g., providing effective feedback to those who report fake news). Drawing on our findings in Sect. 4.2, we argue in Fig. 2 that the impact of fake news can be mitigated by building an integrated approach that combines a tripartite strategy from an individual, institutional and platform level.

### Limitations and future research directions

In this study, we focused on sources in general, which means further research is required to explore the roles played by different sources (e.g., traditional A-list celebrities vs. micro-celebrities vs. family and friends) with different degrees of psychological closeness and credibility. Furthermore, the study sample comprised respondents from different ethnic backgrounds, gender and religion. However, this study did not specifically investigate the effect of, for example, religion, ethnicity or disability on social media users’ susceptibility to fake news. Therefore, future research could explore the role of religion and ethnicity on social media users’ susceptibility to fake news. Furthermore, while it was implied that the age of a social media user plays a role in public susceptibility to fake news, there was no clear understanding of which age group is more likely to believe and share fake news. This could also be an area for future research to better understand the dynamics between age groups vis-à-vis fake news believability and dissemination. Additionally, this study explored the factors that influence public susceptibility to fake news and strategies that can be used to mitigate its impact. Hence, future research can use an experimental design to investigate the effectiveness of some of the strategies identified (e.g., mindfulness-based interventions and digital resilience on consumers’ susceptibility to fake news) in this research. Finally, given the qualitative nature of the study, these findings are specific to the current research context and may not be statistically generalisable.

## Figures and Tables

**Table 3:**
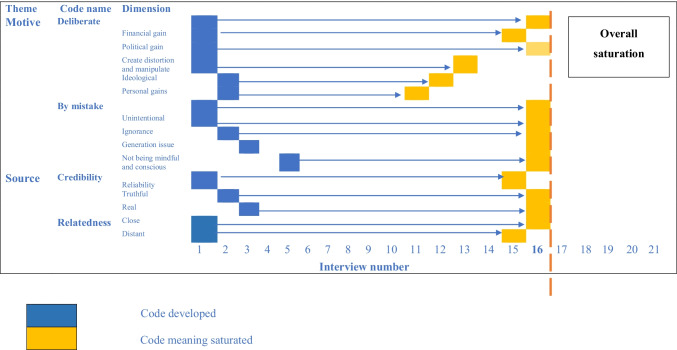
Timing of code development versus code and meaning **saturation (**Example)

## Interview guide

Social media engagement and news exposed or shared during COVID 19.

1. Are you actively using social media?
2. Which social media platforms do you normally use?
3. What type of information do you normally share?
4. Do you share news information on social media?
5. What type of news did you see/receive on social media during COVID 19?
6. How do you assess the credibility of the news that you receive?

Awareness of fake news.

1. With COVID 19, several rumours and fake news were travelling across the globe. Have you experienced this? (Probe: any examples?).
2. Are you aware of different types of fake news shared during COVID -19? (Probe: any examples?).

Dissemination process.

1. How do you think fake news such as those that were shared during COVID-19 is normally spread?
2. Do you think people/organisations share fake news **deliberately** or **mistake**?
3. Why do you think they share them?
4. Who would you attribute the blame to for sharing such fake news?

Strategies to reduce susceptibility to fake news.

1. In your opinion, what are the negative outcomes of fake news?
2. What sort of impact does it have on you and society?
3. What can we do to reduce the spread of fake news?

Demographic information.

1. Age
2. Gender
3. Occupation

## Appendix 2

Tables 2, 3 and 4.

**Table 2:** Timing of code development

Interview number	Number of new codes identified
1	12
2	9
3	4
4	4
5	2
6	5
7	3
8	4
9	3
10	2
11	3
12	3
13	3
14	1
15	1
**16**	**0**
17	**0**
18	**0**
19	**0**
20	**0**
21	**0**

## Appendix 4

**Table 4:** Illustrative sample quotes to support saturation codes and meaning

*Theme*	*Code*	*Sample quotes*
MOTIVE	*Deliberate -Political*	*I can’t help it but I when hear the word fake news, I think immediately about some political leaders who were major proponents in terms of spinning the media to debate the message that want to promote. So, its deliberate and mostly by politicians (RP-1)* *I think, it done by politicians on purpose for their political gains (RP-3)* *I think its deliberate and mostly done for political reasons (RP5)* *They do it [spread fake news] for political reasons (RP-15)* *It might be a group of people, politicians who are looking for their personal benefits (RP-16)*
	*Deliberate – Ideological gains*	*I would like to believe that this could be deliberate or more as a mischief and nonintentional. There is also an ideological angel to this. In any case, I believe it’s planned and deliberate most of the times (RP-2)*
	=	*I would say they do it by default because of their underlying ethos, beliefs and motives (RP-1)* *I think it’s just certain people do it deliberately, who try to put their ideologies to other people to believe. Most of the time they (fake news spreaders) are trying to control other people’s mind nonauthentic way (RP-3)* *Some people and organisations do it to promote their own ideologies (RP5)*
	*Deliberate -Financial and personal gains*	*When fake news comes from some specific organisation, some specific pages or some specific people, they do it deliberately. There are two groups involved. One is the group that aim to use fake news to generate money, to generate profit or generate specific benefit for themselves by manipulating people *via* spreading fake news to change people’s mindset. Such people want to take advantage of people and they know they are doing something wrong, but they are just greedy and do not care about others except themselves (RP-13)* *I am convinced they do it deliberately. I supposed someone who is just forwarding messages they got from elsewhere could be doing it innocently but when you think about these gossip papers who originated the news, they do it deliberately (RP14)*
	*Deliberate/Personal gains (manipulate others)*	*It is either they are trying to scare people or trying to change a given narrative or they have got some sort of hidden personal and or political agenda (RP-5)* *It may be that they are sharing it because it benefits them through more traffic on their website and more traction for their names. I think if a company or organisation is involved in this, it is deliberately but not so much for the individuals who are taking information from these big sources. (RP-9)* *However, I do believe that sometimes people release information to confuse and manipulate individuals about an event, which then you would classify as fake news. But we have to appreciate that not everyone looks into the news they share in-depth, and this makes it easy for fake news to spread online (RP-12)* *Fundamentalists try to use the wave of fake news to manipulate people in different occasions (RP-13)*
	*By mistake Due to ignorance* *Deliberate -personal gains*	*There are some people that share fake information and don’t really know or not critically look at the credibility of the sources. You end up having many people receiving, forwarding, receiving, and sharing without really knowing if such information is fake or true. (RP-10)* *But the creators of such content know that this is fake, and they are mischievous people who wants to build a certain agenda with an ulterior motive. So, while the sharers may spread fake news ignorantly, the creators do so deliberately (RP-10)*
	*Deliberate* *By mistake*	*When fake news comes from some specific organisation, some specific pages or some specific people, they do it deliberately (RP-13)* *But for a large number of people, they just share fake news without having a deeper understanding of the authenticity and impact of falsehood. So, there are two groups involved. One is the group that aim to use fake news to generate money, to generate profit or generate specific benefit for themselves by manipulating people *via* spreading fake news to change people’s mindset (RP-13)* *It could be that people share by mistake and they could be innocent (RP-16)* *But as a marketeer myself, I really know that there's a reason behind every share especially by the people that I follow on social media. They share things with a reason. If they share some raw information, there's a reason behind of it as well. And most if it is because it's for their own personal benefit, I think (RP16)*
	*Source credibility* *Source credibility -Known/related* *Source -Close related /friends and family* *Source credibility-reliable/Trustworthy* *Source- credibility; Closely related* *Source- Trustworthy- Closely related* *Source -Trustworthy -Reliable -Someone related/known*	*The issue of who owns a source and their original intention becomes very critical in this sense (RP-1)* *One of the things I look at for when I come across information is to look at the source even before I start reading. So, if the source is suspicious, I don’t even bother reading it (RP2)* *it comes from a recognisable source like the Independent or BBC, then I more likely to believe it (RP9)* *I think it is also useful to double check with the person purporting to share or send you a piece of information because I have noticed a series of fake account recently on Fake news pretending to be my friend and sending me all sort of dodgy information (RP-10)* *I would consider for credibility is probably the way they share the story. It also depends on who shared this story. I mean if this person is someone I have been following on social media for years, then a certain trust and a relationship develops between us. This could be a measure of how I trust the source (RP-16)* *But within my families, there were people that share absolutely everything and anything, and they don't check the authenticity of it, including my own mum as she worries about me too much. So sometimes the stuff she shares are not neutral. But even considering all of this, I trust what they shared the most than influencers and an absolute stranger (RP-16)* *Sharers believe and trust the original source, the just go ahead and circulate without verifying with the mindset that their close contacts cannot mislead them or cannot be in the wrong (RP-8)* *Sometimes such information come from pretentious sources by mimicking credible sources known to us (RP-10)* *A lot of times if I see a news article of on a platform that isn't like a reliable source, which I would say is like a news outlet such as BBC News or Sky News, then I will like to look at it, read it, and then go look to see if I could find same on another platform (RP-12)* *It also depends on who shared this story. I mean if this person is someone I have been following on social media for years, then a certain trust and a relationship develops between us. This could be a measure of how I trust the source (RP-16)* *I normally follow people I find reliable, mostly my close friends or someone trustworthy. If they share something, I find it credible (RP-18)* *If the news comes from someone I know. I trust it and I think its reliable (RP21)*
